# Changes and Resignations

**Published:** 1881-09-20

**Authors:** 


### EDITORIALS AND MISCELLANEOUS.

### *CHANGES AND RESIGNATIONS.*

DR. JOHN G. WESTMORELAND has resigned the chair of Materia Medica, so long and ably held in the Atlanta Medical College, and Dr. J. S. Todd has been elected to supply the vacancy. *W.*

DR. H. F. SCOTT, the intelligent Professor of the Eye, Ear and Throat in the Southern Medical College has resigned his place, and Dr. A. G. Hobbs has been elected in his stead. Dr. Hobbs is a specialist of experience in this department, has had fine educational advantages and is eminently qualified for the position. W.

THE chair of Diseases of Children, in the Southern Medical College, so ably filled by our co-editor and friend, Dr. W. T. Goldsmith, has been made vacant by his resignation. The duties of this chair will hereafter attach to Dr. Thomas S. Powell, Professor of Obstetrics, whose experience in this branch, and whose ability as a lecturer, give ample assurance that the department will be well and thoroughly taught. *W.*

DR. W. G. OWEN, of Atlanta, Lecturer on Nervous Diseases, has been elected to the chair of Practice in the Southern Medical College, made vacant by the resignation of our distinguished friend, Dr. Alban S. Payne, of Virginia. While the Faculty regret the departure of Dr. Payne, they are pleased to state that his mantle has fallen upon worthy shoulders, Dr. Owen being an experienced physician and a lecturer of rare ability. W.

